# PTEN deficiency sensitizes endometrioid endometrial cancer to compound PARP-PI3K inhibition but not PARP inhibition as monotherapy

**DOI:** 10.1038/onc.2017.326

**Published:** 2017-09-25

**Authors:** X Bian, J Gao, F Luo, C Rui, T Zheng, D Wang, Y Wang, T M Roberts, P Liu, J J Zhao, H Cheng

**Affiliations:** 1 Cancer Institute, The Second Hospital of Dalian Medical University, Institute of Cancer Stem Cell, Dalian Medical University, Dalian, China; 2 Department of Acute Abdomen Surgery, The Second Hospital of Dalian Medical University, Dalian, China; 3 College of Basic Medical Sciences, Dalian Medical University, Dalian, China; 4 Department of Obstetrics and Gynecology, The Second Hospital of Dalian Medical University, Dalian, China; 5 Department of Cancer Biology, Dana-Farber Cancer Institute, Boston, MA, USA; 6 College of Pharmacy, Dalian Medical University, Dalian, China

## Abstract

Poly (ADP-ribose) polymerase (PARP) inhibitors have emerged as promising cancer therapeutics especially for tumors with deficient homologous recombination (HR) repair. However, as HR-deficient tumors represent only a small fraction of endometrial cancers, the therapeutic utility of PARP inhibitors is limited in this disease. Somatic loss of phosphatase and tensin homolog (PTEN), a tumor suppressor that counteracts phosphoinositide 3-kinase (PI3K) activity, is one of the most common genetic aberrations in endometrioid endometrial cancer. While previous works have identified the role of PTEN in DNA double-strand break repair, vulnerabilities of PTEN-deficient endometrioid endometrial cancers to PARP inhibition remain controversial. Here we find that PTEN-deficient endometrioid endometrial cancer cells are not responsive to PARP inhibitor Olaparib alone, but instead show superior sensitivity to compound inhibition with PI3K inhibitor BKM120, as evidenced by reduced clonogenic cell growth and three-dimensional (3D) spheroid disintegration. Mechanistically, PI3K blockade by BKM120 attenuated HR competency with γH2AX accumulation and reduced RAD51 and BRCA1 expression in Ishikawa, AN3CA and Nou-1 cells, but the same combination treatment led to enhanced phosphorylation of DNA-PK, a non-homologous end joining repair protein, in Hec-108 cells. Furthermore, we show that CRISPR/Cas9-mediated *PTEN* depletion rendered PTEN wild-type Hec-1A endometrioid endometrial cancer cells responsive to combined inhibition of PARP/PI3K, with concomitantly induced DNA damage accumulation and repair defects. The combination of BKM120 and Olaparib cooperated to inhibit tumor growth in a genetic mouse model of *Pten*-deficient endometrioid endometrial cancer. Together, these results suggest PI3K inhibition may be a plausible approach to expand the utility of PARP inhibitors to endometrioid endometrial cancers in a PTEN-deficient setting.

## Introduction

Endometrial cancer is the most common gynecological malignancy among women in the developed country. It can be broadly classified into two types: majority (~80%) of endometrial cancers are of Type I endometrioid histology; up to 15% are of Type II, primarily serous carcinomas.^1, 2^ Most of endometrioid endometrial cancer patients present with low-grade and early-stage disease and have a favorable prognosis. However, treatment options for metastatic or late-stage endometrioid endometrial cancer are limited and the outcome is extremely poor.^3^ There is thus a pressing need to develop novel effective therapeutics for advanced endometrial cancer to improve patient outcomes.

Olaparib, a poly (ADP-ribose) polymerase (PARP) inhibitor (PARPi), has been approved as the first ‘personalized therapy’ for advanced *BRCA1/2* mutated ovarian cancer.^4^ However, unlike ovarian cancers with nearly half of the cases bearing deficiency in homologous recombination (HR),^5^ majority of endometrial cancers harbor intact HR pathway, which thus limits the therapeutic utility of PARP inhibitors in this disease. Olaparib and other PARP inhibitors as monotherapy or in combination therapies are being actively assessed in the treatment of a variety of cancer types bearing deficient BRCA, including ovarian cancer, prostate cancer and breast cancer.^6, 7, 8, 9^ Meanwhile, recent studies reveal that the concept of synthetic lethality to target non-*BRCA*-mutant cancers with PARP inhibitors also has clear potential.^10, 11, 12^

Somatic loss of phosphatase and tensin homolog (*PTEN*) is one of the most common genomic aberrations in endometrioid endometrial cancer.^2, 13^ The tumor suppressor PTEN antagonizes the activation of the phosphoinositide 3-kinase (PI3K)/AKT pathway, a cell pro-survival signaling cascade important for the initiation and maintenance of endometrial cancer.^3, 14, 15^ Meanwhile, PTEN also plays tumor-suppressive roles in the nucleus by maintaining genome integrity.^16, 17, 18^ Loss of PTEN impairs CHK1 function, leading to accumulation of DNA double strand breaks and genomic instability.^16^ PTEN regulates the expression of RAD51, a key protein in HR repair of DNA double strand breaks.^17^ PTEN deficiency has thus been proposed to be predictive of sensitivity to PARP inhibitors.^18, 19^ However, there are conflicting results regarding the synthetic lethal targeting of *PTEN*-deficient endometrial cancer cells with PARP inhibitors.^20, 21, 22^ Furthermore, treatment response to PARP inhibitors has not been extensively examined in genetic mouse models of endometrioid endometrial cancer in the setting of PTEN loss.^23^

The discovery that the PI3K pathway is among the most frequently dysregulated signaling networks in human malignancies has made it a major target for cancer treatments.^14, 24^ However, recent clinical trials of pan-PI3K inhibitors have not yet yielded exciting results.^25, 26^ To improve clinical efficacy of PI3K inhibitors (PI3Ki), combination therapies are being actively developed and evaluated in preclinical and clinical settings for the treatment of cancers harboring hyperactivated PI3K signaling pathway.^27^ PTEN-deficient tumor cells have been found sensitive to PI3K inhibition in combination with genotoxic stress (irradiation or cisplatin) both *in vitro and in vivo*.^28^ We and others have recently reported the therapeutic efficacy of combined use of PI3K inhibitor BKM120 and PARP inhibitor Olaparib for cancer treatments, including both BRCA1-proficient and -deficient breast cancer,^29^ PTEN-deficient prostate cancer,^30^ as well as ovarian cancer^31, 32^ and lung cancer.^33^ Meanwhile, the therapeutic strategy of using combined BKM120/Olaparib is being tested in a Phase I clinical trial in patients with recurrent triple negative breast cancer and high-grade serous ovarian cancer (www.clinicaltrials.gov). However, such drug combination has not been investigated in the setting of endometrioid endometrial cancers in which majority of the cases harbor PTEN deficiency. In the current study, we tested whether single-agent Olaparib or in combination with BKM120 would represent effective treatment modalities for PTEN-deficient endometrioid endometrial cancer.

## Results

### Combined inhibition of PARP and PI3K cooperates to inhibit the growth of PTEN-deficient endometrioid endometrial cancer cells *in vitro*

**Figure 1:**
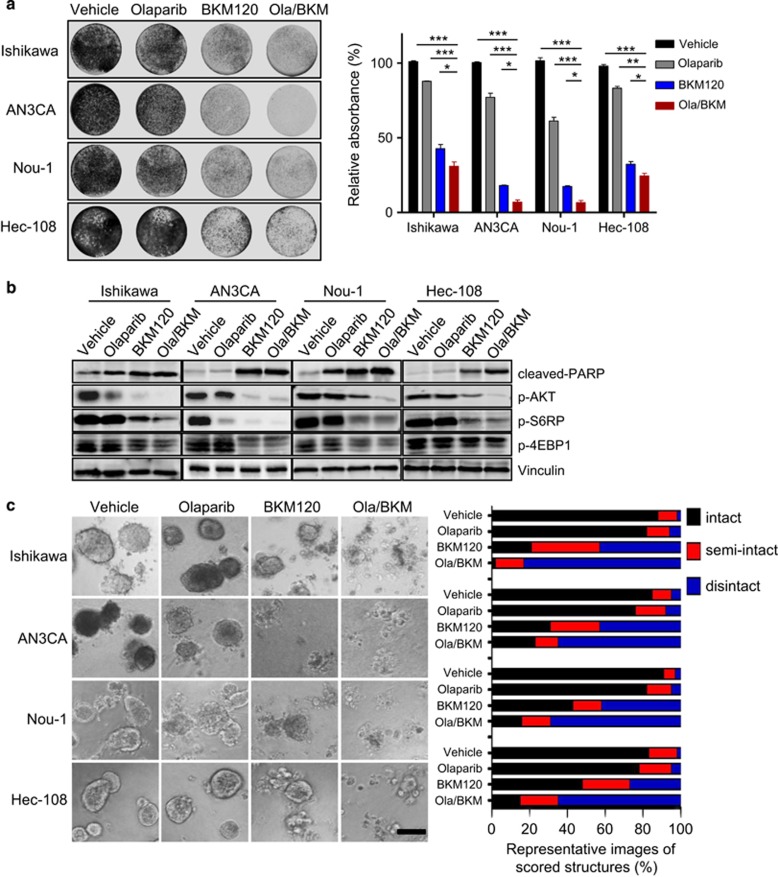
Effects of Olaparib and BKM120 as single-agents or in combination on the survival and growth of PTEN-deficient endometrioid endometrial cancer cells. (**a**) PTEN-deficient endometrioid ensdometrial cancer cell lines as indicated were treated with drugs for 7–10 days. Fresh media with drugs were replaced every 3 days. At the end point, plates were fixed and stained with crystal violet. All experiments were performed in triplicate. Representative images of plates are shown. Error bars represent standard deviations (s.d.) from the mean. *P<0.05; **P<0.01; ***P<0.001 (Student’s *t*-test). (**b**) PTEN-deficient endometrioid endometrial cancer cell lines were treated with drugs as indicated for 24 h. Phosphorylated AKT, S6RP and 4EBP1 proteins and cleaved PARP were detected by western blot. Vinculin served as a loading control. (**c**) PTEN-deficient endometrioid endometrial cancer cell lines were cultured in 3D matrigel and drug treated for 10–12 days. Representative images of cells are shown in the left panel. Quantification of scored structures (intact, semi-disintegrated and disintegrated) is shown in the right panel. Scale bar, 100 μm.

To investigate how endometrioid endometrial cancer cells respond to PARP inhibition, we treated several endometrioid endometrial cancer cell line models that lack PTEN (Ishikawa, AN3CA, Nou-1, and Hec-108) with PARP inhibitor Olaparib as single-agent or in combined with BKM120, a pan-class I PI3K inhibitor. The Olaparib/BKM120 combination demonstrated synergistic growth inhibition of all four PTEN-deficient endometrioid endometrial cancer cell lines examined (Supplementary Figure 1). In the clonogenic assay, we found that Olaparib alone showed little inhibitory effect on the growth of PTEN-deficient endometrioid endometrial cancer cells (Figure 1a). In contrast, BKM120 as single-agent, and to a more significant extent in combination with Olaparib, led to remarkably attenuated growth of PTEN-deficient endometrioid endometrial cancer cells. As expected, PI3K inhibition by BKM120 alone, and to a remarkable extent, in combined with Olaparib, led to diminished phosphorylated AKT (p-AKT) signals in all four endometrioid endometrial cancer cell lines examined (Figure 1b). In accord, p-S6RP and p-4EBP1 signals, effectors downstream of AKT/mTOR signaling, were also pronouncedly downregulated. We also noticed a consistent and substantial increase in the abundance of cleaved PARP protein, an indication of enhanced apoptosis, in cells treated with BKM120 alone and in combination with Olaparib (Figure 1b).

To assess the drug effects in conditions that more closely mimic tumor microenvironment, we subjected endometrioid endometrial cancer cells to culture as 3D spheroids in matrigel. We found that PTEN-deficient endometrioid endometrial cancer cells cultured under 3D condition also did not respond to PARP inhibition by Olaparib (Figure 1c). While PI3K inhibitor BKM120 as single-agent induced spheroid disintegration to a pronounced degree, combination of Olaparib and BKM120 led to a more substantial structural disintegration in all four PTEN-deficient endometrioid endometrial cancer cell lines examined (Figure 1c). Together, these data suggest combined inhibition of PARP and PI3K may be an effective treatment strategy for PTEN-deficient endometrioid endometrial cancer cells.

### Olaparib and BKM120 cooperate to trigger DNA damage via differential mechanisms in PTEN-deficient endometrioid endometrial cancer cells

**Figure 2:**
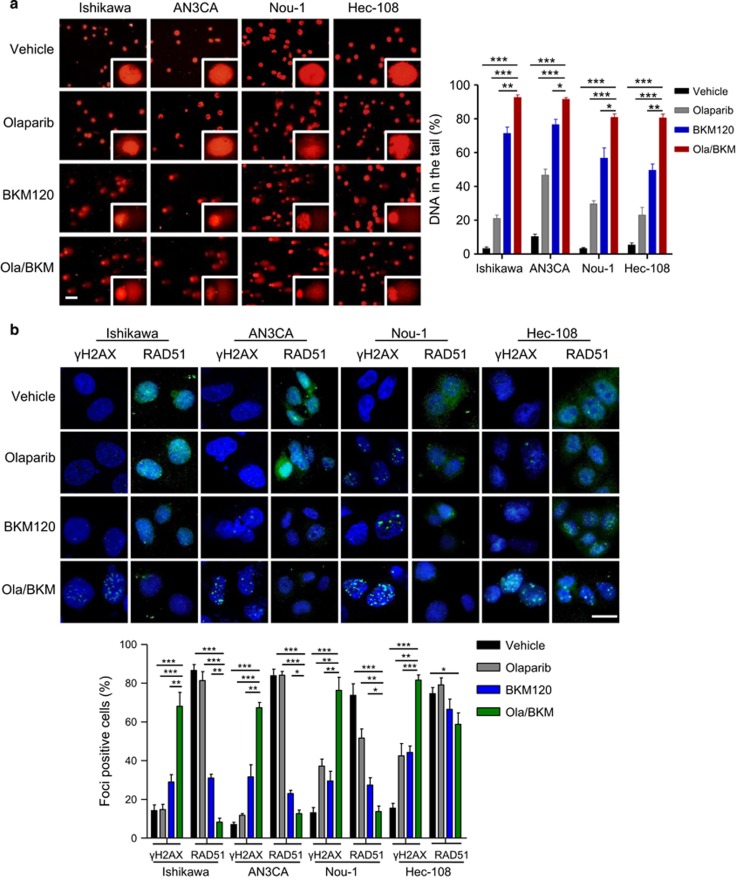

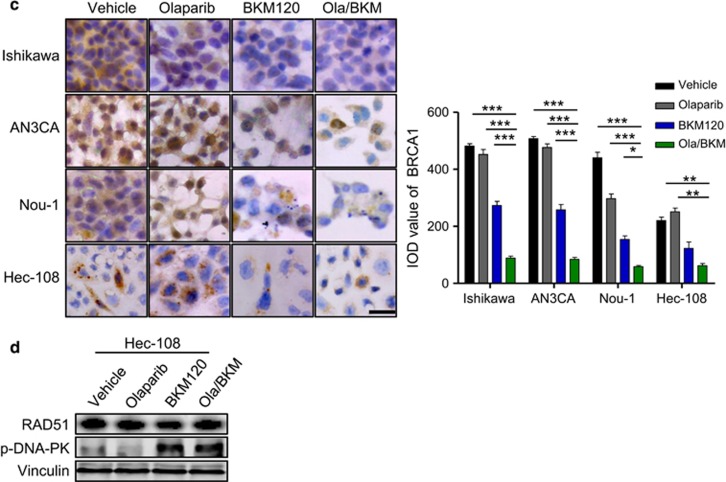
Effects of Olaparib and BKM120 as single-agents or in combination on DNA damage and repairs in PTEN-deficient endometrioid endometrial cancer cells. (**a**) DNA damage was measured by comet assay in PTEN-deficient endometrioid endometrial cancer cells treated with drugs as indicated for 48 h. Scale bar, 50 μm. Quantification of DNA in the tail from three independent experiments is shown as mean±s.d. (**b**) Immunofluorescent staining of RAD51, γH2AX and DAPI in respective PTEN-deficient endometrioid endometrial cancer cells treated with drugs as indicated for 48 h. Scale bar, 20 μm. Cells containing more than five foci were scored as positive. Means±s.d. for three independent experiments are shown. (**c**) Representative images of immunocytochemical staining analyses of BRCA1 protein in PTEN-deficient endometrioid endometrial cancer cells treated with drugs as indicated for 48 h. Scale bar, 50 μm. Quantification of IOD (integrated optical density) value of BRCA1 from three independent experiments is shown as mean±s.d. (**d**) Western blot analysis of phosphorylated-DNA-PK in Hec-108 endometrioid endometrial cancer cells treated as indicated for 24 h. Vinculin served as a loading control. *P<0.05; **P<0.01; ***P<0.001 (Student’s *t*-test).

To understand the molecular mechanism underlying the superior sensitivity to dual PARP/PI3K inhibition, we first assessed DNA damage by comet assays. Whereas PARP inhibitor Olaparib alone did not elicit prominent DNA damage in any of the PTEN-deficient endometrioid endometrial cancer cell lines examined, the PI3K inhibitor BKM120 as single-agent, and to a more significant extent in combination with Olaparib, induced robust amounts of DNA in the tails, indicating DNA strand breaks (Figure 2a). In accord with this, we observed an accumulation of γH2AX nuclear foci, a surrogate marker for DNA double strand breaks in Olaparib/BKM120-treated cells (Figure 2b). Additionally, all four PTEN-deficient endometrioid endometrial cancer cell lines revealed significant increase in the formation of aberrant chromosome structures after combined treatment with Olaparib and BKM120 when compared to those in vehicle or single-agent treatment groups (Supplementary Figure 2). Together, these results suggest that PI3K blockade may induce DNA repair defects and thus cause synthetic lethality upon PARP inhibition in PTEN-deficient endometrioid endometrial cancer cells.

We noticed that PTEN-deficient endometrioid endometrial cancer cells still efficiently formed RAD51 nuclear foci (Figure 2b), a crucial component of the HR repair.^34^ Whereas PARP inhibitor Olaparib treatment did not affect the formation of RAD51 nuclear foci, PI3K blockade by BKM120 alone or in combination with Olaparib resulted in a remarkable reduction in RAD51 nuclear foci formation in Ishikawa, AN3CA and Nou-1 cells (Figure 2b). In contrast, dual PARPi/PI3Ki did not have a significant effect on RAD51 nuclear foci formation in Hec-108 cells (Figure 2b). Further analysis of BRCA1, another protein crucial for HR repair,^35^ by quantitative RT–PCR and immunocytochemical staining revealed BKM120 treatment led to reduced BRCA1 mRNA levels and exclusive cytoplasmic localization of BRCA1, respectively (Supplementary Figure 3 and Figure 2c). Together, these results suggested that PI3K inhibition led to impaired competency of HR repair in Ishikawa, AN3CA and Nou-1 endometrioid endometrial cancer cells. Notably, while combined use of PARP/PI3K inhibitors induced accumulation of γH2AX in Hec-108 cells, it had little impact on RAD51 nuclear foci formation (Figure 2b), suggesting a mechanism other than deficiency in RAD51-dependent HR repair may account for the cooperative effect of BKM120 and Olaparib. Autophosphorylation of DNA-PK appears to play a key role in non-homologous end joining pathway of DNA repair.^36^ Indeed, BKM120 treatment resulted in marked induction of phosphorylation of DNA-PK in Hec-108 cells (Figure 2d), suggesting that activation of non-homologous end joining incurred by PI3K inhibition likely confers the cytotoxic effects of Olaparib seen in Hec-108 cells.

### PTEN deficiency predicts sensitivity to the Olaparib/BKM120 combination treatment in endometrioid endometrial cancer cells

**Figure 3:**
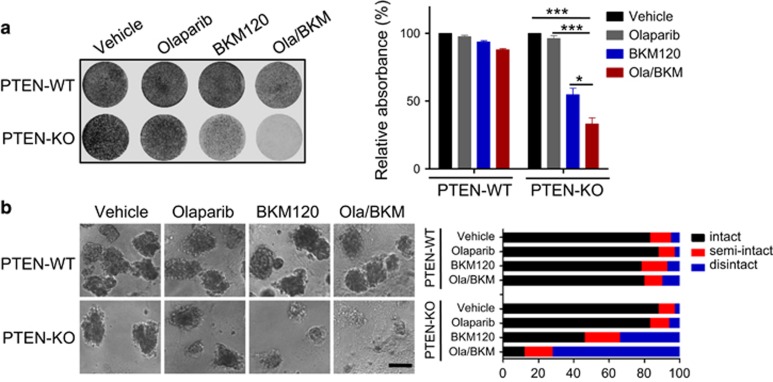
Effects of PTEN loss on sensitivity to the combined Olaparib and BKM120 treatment in Hec-1A endometrioid endometrial cancer cells. (**a**) PTEN-proficient (PTEN-WT) and deficient (PTEN-KO #3) Hec-1A endometrioid endometrial cancer cells were treated with BKM120 and Olaparib as single-agents or in combination for 10 days. Fresh media with drugs were replaced every 3 days. At the end point, plates were fixed and stained with crystal violet. All experiments were performed in triplicate. Representative images of plates are shown. Error bars represent mean±s.d. (**b**) Hec-1A cells (PTEN-WT vs PTEN-KO #3) were cultured in 3D and treated with drugs as indicated for 10 days. Representative images of cells are shown in the left panel. Quantification of scored structures (intact, semi-disintegrated and disintegrated) is shown in the right panel. Scale bar, 100 μm. *P<0.05; ***P<0.001 (Student’s *t*-test).

We next examined if PTEN status may affect the response of endometrioid endometrial cancer cells to Olaparib as single-agent and in combination with BKM120. We first employed the newly developed CRISPR/Cas9 system to deplete PTEN in Hec-1A cells (an endometrioid endometrial cancer cell line with intact PTEN, PTEN-WT), and named them PTEN-KO cells thereafter (Supplementary Figures 4a and b). Consistent with the role of PTEN in antagonizing PI3K signaling, PTEN depletion strongly induced AKT activation and BKM120 as single-agent significantly attenuated PI3K/AKT/mTOR signaling pathway (Supplementary Figure 5). Remarkably, Hec-1A PTEN-KO cells showed striking response to the combined use of Olaparib and BKM120, as shown with significantly reduced clonogenic growth and massive disintegration of 3D spheroids in matrigel (Figures 3a and b). It is worth noting, however, depletion of PTEN in Hec-1A cells had little effect on the response to Olaparib as single agent (Figures 3a and b), similar to the observation with PTEN-deficient endometrioid endometrial cancer cells. Together, these results suggest that PTEN status may affect the response of endometrioid endometrial cancer cells to combined PARPi/PI3Ki but not PARPi alone.

**Figure 4:**
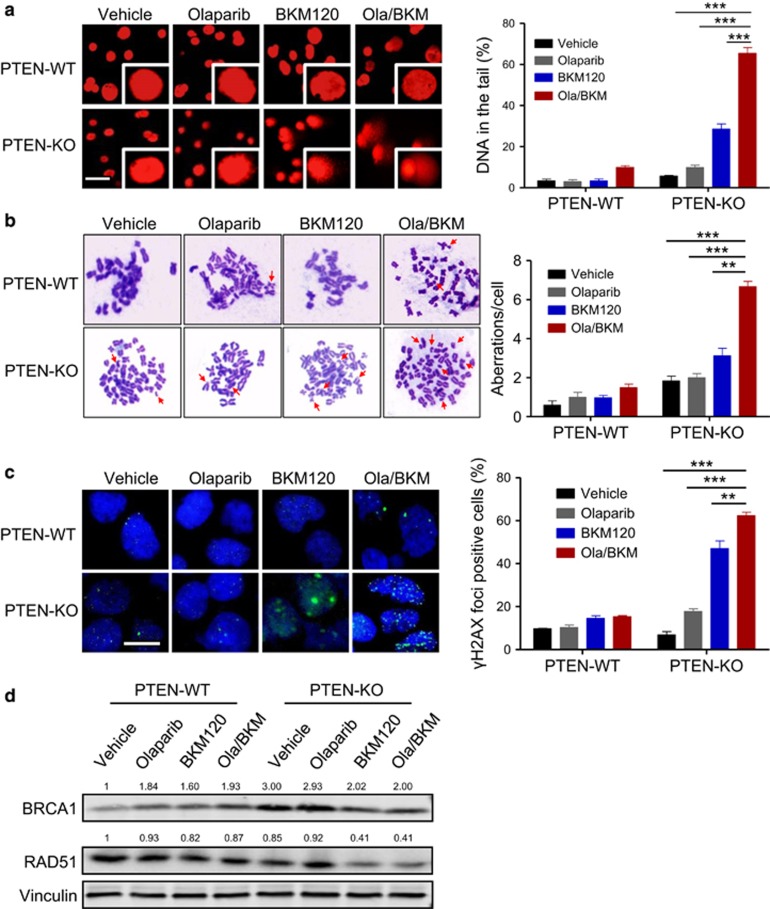
Effects of PTEN loss on DNA damage and repairs in response to the combined Olaparib and BKM120 treatment in Hec-1A endometrioid endometrial cancer cells. (**a**) DNA damage was measured by comet assay in Hec-1A cells (PTEN-WT vs PTEN-KO #3) treated with drugs as indicated for 48 h. Scale bar, 50 μm. Quantification of DNA in the tail from three independent experiments is shown as mean±s.d. (**b**) Metaphase spread analysis of chromosome aberrations in Hec1-A cells (PTEN-WT vs PTEN-KO #3) after drug treatments as indicated for 48 h. Representative metaphase spreads are shown. Arrows indicate chromosomal aberrations. Mean±s.d. for three independent experiments are shown. (**c**) Representative images of immunofluorescent staining of γH2AX and DAPI in Hec1-A cells (PTEN-WT vs PTEN-KO #3) treated with drugs as indicated for 48 h. Scale bar, 20 μm. Cells containing more than five foci were scored as positive. Means±s.d. for three independent experiments are shown. (**d**) Western blot analysis of RAD51 and BRCA1 proteins in Hec1-A cells (PTEN-WT vs PTEN-KO #3) treated as indicated for 24 h. Vinculin served as a loading control. **P<0.01; ***P<0.001 (Student’s *t*-test).

Consistent with our observation with PTEN-deficient endometrioid endometrial cancer cells (Figure 2a), depletion of PTEN in Hec-1A cells did not affect DNA damage upon Olaparib treatment (Figures 4a and b). In contrast, combined use of BKM120 and Olaparib induced significantly elevated levels of DNA strand breaks and the formation of aberrant chromosome breaks in Hec-1A PTEN-KO cells (Figures 4a and b). In accord with this, we found remarkably induced formation of γH2AX nuclear foci in BKM120 single-agent treated cells, and to an even more significant extent, in cells treated with combined PARPi/PI3Ki (Figure 4c). Further quantitative RT–PCR and western blot analyses revealed reduced abundance of HR repair proteins RAD51 and BRCA1 in response to combined PI3Ki/PARPi treatment (Supplementary Figure 6 and Figure 4d). Together, these results suggest that PI3K inhibition compromises HR repair in PTEN-deficient but not PTEN-proficient endometrioid endometrial cancer cells, rendering superior sensitivity to PARP inhibition.

### Combined use of Olaparib and BKM120 effectively treats *Pten/Lkb1*-deficient endometrioid endometrial tumors

**Figure 5:**
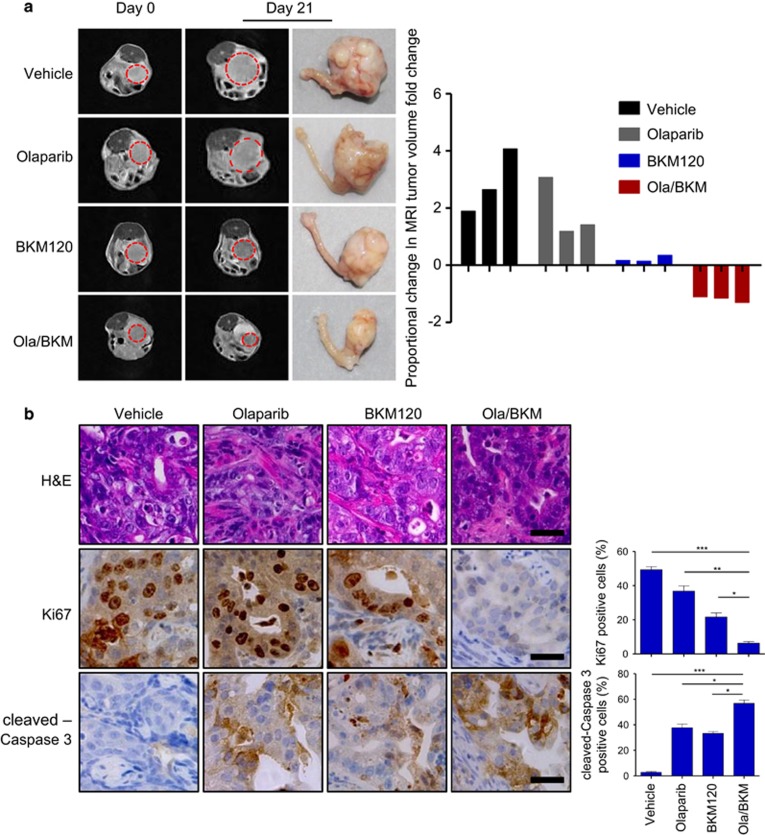

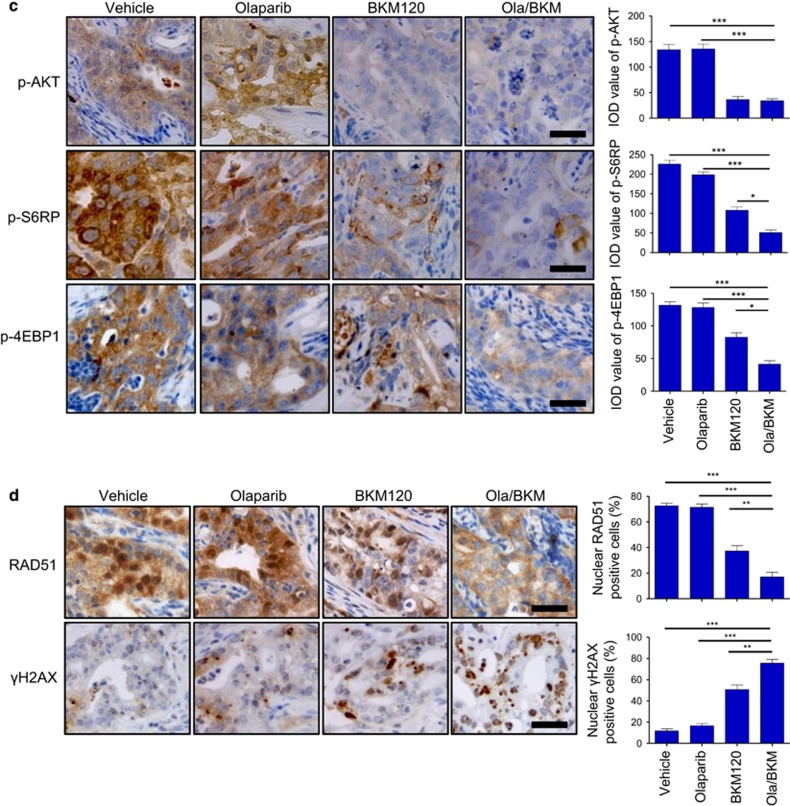
Effects of Olaparib and BKM120 as single-agents or in combination on the genetic mouse model of endometrioid endometrial tumors driven by co-loss of *Pten* and *Lkb1*. (**a**) Female *Pten*^*loxp/loxp*^*/Lkb1*^*loxp/loxp*^ mice were injected with adenovirus expressing Cre recombinase (Ade-Cre). Six weeks post injection, injected mice were treated with Olaparib (50 mg/kg/day, intraperitoneal injection), BKM120 (30 mg/kg/day, oral gavage) as single-agents or in combination. Representative MRI images of mice at initiation (T0) and completion of drug treatment (21 days, T21) (left panel) and the waterfall plot depicting proportional changes in tumor volume (right panel) are shown (*n*=3 per treatment group). Representative images of histological (**b**) and immunohistochemical staining (**b**–**d**) for proteins as indicated in tumors from Ade-Cre-injected *Pten*^*loxp/loxp*^*/Lkb1*^*loxp/loxp*^ mice (*n*=6 per treatment group) treated with BKM120 and Olaparib as single-agents or in combination for 3 days. Scale bar, 25 μm. Data are shown as mean±s.e.m. *P<0.05; **P<0.01; ***P<0.001 (Student’s *t-*test). H&E, hematoxylin and eosin.

We next evaluated the therapeutic efficacy of combining PARP and PI3K inhibitors in a genetic mouse model of *Pten*-deficient endometrioid endometrial cancer established in our laboratory.^37^ This endometrioid endometrial cancer mouse model was generated by conditional ablation of *Pten* and *Lkb1* in mouse endometrial epithelium,^37^ (Supplementary Figure 7). At 6 weeks after injection of adenoviral expressing Cre recombinase (Ade-Cre), mice with similar tumor volumes were treated with Olaparib (50 mg/kg/day), BKM120 (30 mg/kg/day) as single-agents or in combination. None of the treatments caused weight loss in the tumor-bearing mice examined (Supplementary Figure 8). While Olaparib monotherapy exhibited limited efficacy, BKM120 appeared to have a stronger growth inhibitory effect as compared to vehicle treatment (mean fold change in magnetic resonance imaging (MRI) tumor volume increased by 2.87-fold vs 0.22-fold), leading to a stable disease (Figure 5a). In contrast, combined use of Olaparib and BKM120 resulted in strong antitumor efficacy compared with no treatment (mean fold change in MRI tumor volume reduced by 1.83-fold) (Figure 5a). Consistent with the drug effects noted above, we observed significantly reduced Ki67-positive cells and substantially more cleaved-Caspase 3-positive cells in the Olaparib/BKM120 combination treatment group as compared to no treatment or single treatment groups (Figure 5b). Thus, reduced cellular proliferation and increased apoptosis might account for tumor regression seen in mice treated with Olaparib/BKM120. Further immunohistochemical analysis showed nearly completely abolished p-AKT signals, and to a lesser extent p-S6RP and p-4EBP1 signals, in tumors treated with BKM120 alone or in combination with Olaparib (Figure 5c), indicative of target inhibition of PI3K/AKT/mTOR signaling as a result of PI3K inhibitor treatment. Notably, treatment with Olaparib alone did not lead to a discernible change on the activation status of AKT in the PTEN-deficient endometrioid endometrial cancer cell lines examined *in vitro* (Supplementary Figure 9). Nevertheless, we observed markedly induced AKT activation in tumors treated with Olaparib for 10 days (Supplementary Figure 10), indicating that prolonged PARP inhibition as a cellular stress may trigger the activation of the pro-survival PI3K/AKT signaling *in vivo*.^38^ This may partially explain why Olaparib monotherapy is insufficient to achieve a significant anti-tumor effect in PTEN-deficient endometrioid endometrial cancers. Furthermore, Olaparib/BKM120 treatment led to a substantial increase in γH2AX nuclear foci formation while markedly reduced RAD51 nuclear foci (Figure 5d), indicative of accumulation of double-strand DNA breaks as a result of compromised HR repair. Together, the combined treatment of Olaparib and BKM120 demonstrated significant anti-tumor activity in the genetic mouse model of *Pten/Lkb1*-deficient endometrioid endometrial cancer.

## Discussion

There is a pressing need to develop novel effective therapeutics for advanced endometrial cancer. PTEN loss is one of the most common genetic aberrations in endometrioid endometrial cancer. While previous studies have implicated the role of PTEN in HR repair, it remains controversial as to whether cancer cells with PTEN deficiency are vulnerable to PARP inhibitor treatment. In this study, using cancer cell line models and a preclinical genetic mouse model of endometrioid endometrial cancer, we show that PTEN-deficient endometrioid endometrial cancer cells are not responsive to the single treatment with PARP inhibitor Olaparib, but combined inhibition of PARP and PI3K results in a synergistic growth inhibitory effect *in vitro* as well as a cooperative antitumor effect *in vivo*.

It has been previously reported that PTEN contributes to double strand break repair through transcriptional regulation of an essential HR repair protein RAD51 in PTEN-null mouse embryonic fibroblasts,^17^ and that PTEN loss is associated with RAD51 downregulation and PARP inhibitor sensitivity in endometrioid endometrial cancer cells.^20, 21^ Controversially, two recent studies reported that PTEN loss does not affect RAD51 expression or sensitivity to PARPi treatment in preclinical models of prostate cancer and endometrial cancer.^22, 39^ In our study, we also observed reduced RAD51 protein abundance in endometrioid endometrial cancer cells when PTEN is depleted. Together with our observations in PTEN-deficient endometrioid endometrial cancer cell line models, we argue that reduced RAD51 protein abundance/function upon PI3K inhibitor treatment may lead to compromised HR repair and thus account for superior sensitivity to PARP inhibitors in PTEN-deficient endometrioid endometrial cancer cells. It is also worth noting that despite the fact that similar cell line models of PTEN-deficient endometrioid endometrial cancer were examined (^20, 21, 22^ and the current study), differential mechanisms of action for each PARPi (that is, PARP catalytic inhibition vs PARP trapping) used in these studies may contribute to the discrepant results. Further studies are necessary to thoroughly interrogate the complex relationship between PTEN status and the activities of specific PARP inhibitors tested in clinical trials for advanced endometrioid endometrial cancer.

Our results showed that Olaparib treatment on its own is not effective in endometrioid endometrial cancer cells with PTEN deficiency. One explanation for this is that Olaparib may trigger the activation of PI3K/AKT signaling pathway to promote cell survival.^14, 24, 40, 41^ In our study, depletion of PTEN in Hec-1A cells led to superior activation of the pro-survival oncogenic PI3K/AKT signaling. We also observed a significant induction of AKT activation in the genetic mouse model of *Pten*-deficient endometrioid endometrial cancer after prolonged treatment (10 days) with Olaparib (Supplementary Figure 10), thus partially explaining the limited efficacy of PARP inhibitor monotherapy. Similar observations have been reported by recent preclinical studies in which PARPi treatment alone led to an activation of PI3K/AKT signaling, and thus was insufficient to abrogate the growth of prostate and breast tumors.^30, 38^ Together, these results indicate that activation of pro-survival PI3K/AKT signaling may compromise the efficacy of PARPi, raising the caution of PARP inhibitor monotherapy in the treatments of endometrioid endometrial cancer.

In this study, we show that PI3K inhibition by BKM120 treatment efficiently blocks AKT activation and elicits DNA damage upon PARP inhibition in PTEN-deficient endometrioid endometrial cancer cells as well as in an isogenic cell line model with PTEN loss. The combinatorial treatment with BKM120 and Olaparib resulted in a synergistic growth inhibitory effect *in vitro*, as evidenced by reduced proliferation of cells growing in monolayer, and disintegration of 3D spheroids in a more ‘tumor-like’ setting. Moreover, whereas BKM120 monotherapy moderately attenuated tumor growth, combinatorial inhibition of PI3K and PARP significantly regressed *Pten*-deficient endometrioid endometrial tumors as assessed by MRI analysis. Thus, our results suggest that the PI3Ki/PARPi combination may represent an effective therapeutic approach in PTEN-deficient endometrioid endometrial tumors. PARP inhibitors have emerged as promising cancer therapeutics especially for cancers that are deficient in HR repair.^42, 43^ Our findings may have the potential to extend the therapeutic utility of PARP inhibitors into a broader population of patients suffering from this disease.

## Materials and methods

### Cell culture and reagents

Ishikawa, AN3CA, Nou-1, Hec-108 and Hec-1A human endometrioid endometrial cancer cell lines were obtained from Dana-Farber Cancer Institute, Harvard Medical School. All cells were examined periodically for Mycoplasma infection. Cells were maintained in culture medium (Ishikawa cells in Dulbecco's Modified Eagle Medium; AN3CA, Nou-1 and Hec-108 cells in RPMI-1640; Hec-1A cells in McCoy’s 5A) supplemented with 10% fetal bovine serum and penicillin/streptomycin (100 units/ml) at 37 °C and 5% CO_2_. The pan-PI3K inhibitor NVP-BKM120 was purchased from Biochempartner (Shanghai, China) and PARP inhibitor Olaparib (AZD2281) was purchased from Chemexpress (Shanghai, China). sgRNA sequences targeting PTEN (#1: 5′-TTATCCAAACATTATTGCTA-3′ #2: 5′-CCTACCTCTGCAATTAAATT-3′ #3: 5′-ACCGCCAAATTTAATTGCAG-3′) were cloned into the lentiCRISPR v1 (#49535, Addgene, Cambridge, MA, USA).

### Clonogenic assay

Cells were seeded on plates and cultured for 24 h before the initiation of drug treatment. Fresh media containing drugs was replaced every 3 days. At the end point, cells were washed with phosphate buffered solution and fixed with fixation solution and subsequently stained with 5% crystal violet solution. Images of stained plates were captured using Molecular Imager (Bio-Rad Laboratories, Hercules, CA, USA). The optical absorbance of bound crystal violet (dissolved in 10% acetic acid) was measured at 570 nm by Multi-functional microplate reader Enspire230 (PerkinElmer, Waltham, MA, USA).

### 3-dimensional sphere culture

Three-dimensional sphere culture experiments were performed as previously described.^44^ Endometrioid endometrial cancer cells were seeded on plates with 50% precoated matrigel (BD Biosciences, San Jose, CA, USA) plus 50% of medium without serum. Cells were cultured in media supplemented with 5% fetal bovine serum and 2% matrigel that was replaced every 3 days. Three-dimensional cultures were imaged by inverted phase contrast microscope (Leica Microsystems, Wetzlar, Germany) and scored according to 3D structure integrity. Over 100 structures were scored for each type of drug treatment.

### Immunoblotting analysis and antibodies

Cell lysate was prepared using RIPA buffer supplemented with protease/phosphatase inhibitors. Immunoblotting experiment was conducted as described previously.^37^ The following primary antibodies were used: p-AKT Ser473 (1:800, 4060, Cell Signaling Technology, Beverly, MA, USA), p-S6RP Ser235-/236 (1:1000, CST 4858), p-4EBP1 Thr37/46 (1:1000, CST 2855), cleaved-PARP (1:1000, CST 9546), PTEN (1:200, CST 9559), BRCA1 (1:1000, 22362-1-AP, Proteintech, Rosemont, IL, USA), RAD51 (1:500, sc-8349, Santa Cruz Biotechnology, Santa Cruz, CA, USA), p-DNA-PK (S2056, ab124918, Abcam, Cambridge, MA, USA) and Vinculin (1:10000, V9131, Sigma-Aldrich, St Louis, MO, USA).^10, 37, 38, 45^ Immunofluorescently labeled secondary antibodies to rabbit-IgG (Molecular Probes, Grand Island, NY, USA) or mouse-IgG (Rockland Immunochemicals, Limerick, PA, USA) was used. Western blots were imaged with LI-COR Odyssey (LI-COR Biosciences, Lincoln, NE, USA).

### Comet assay and Metaphase chromosome spread assay

A comet assay was performed as previously stated^31^ and 200 randomly selected cells were analyzed using Casplab software. The level of DNA damage was presented as percentage of DNA in tail. For metaphase chromosome spread assay, cells were incubated with colchicine (0.5 μg/ml) for 4 h and metaphase spreads were prepared as described previously.^46^

### Quantitative RT–PCR

Total RNA from endometrioid endometrial cell lines was isolated using Trizol (Life Technologies, Grand Island, NY, USA). Reverse transcription reaction was performed using PrimeScript RT Master Mix kit (Takara, Dalian, China). Quantitative PCR was conducted using SYBR Green PCR master Mix (Life Technologies) on Mx3005P real-time PCR system. BRCA1 was amplified with primers, Fw 5′-GTCCCATCTGTCTGGAGTTGA-3′, Rv 5′-AAAGGACACTGTGAAGGCCC-3′. BRCA2 was amplified with primers, Fw 5′-TGCCTGAAAACCAGATGACTATC-3′, Rv 5′-AGGCCAGCAAACTTCCGTTTA-3′.

### Immunofluorescent staining

Immunofluorescent staining was conducted as described.^11^ The primary antibodies γH2AX (1:800, Ser139, CST 2577), RAD51 (1:200, Santa Cruz sc-8349)^32, 47^ and fluorescence-conjugated secondary antibodies were used. Images were captured with a Leica fluorescence microscope (Leica Microsystems).

### Histology and immunohistochemical staining

Formalin-fixed and paraffin-embedded blocks were prepared as described previously.^37^ Sectioned paraffin blocks were stained with hematoxylin and eosin (H&E) for histological analysis. The following primary antibodies were used for immunohistochemical staining: p-AKT Ser473 (1:200, 785697A, Invitrogen, Carlsbad, CA, USA), p-S6RP Ser235/236 (1:400, CST 4858), p-4EBP1 Thr37/46 (1:1000, CST 2855), RAD51 (1:200, Santa Cruz sc-8349), γH2AX (1:500, Ser139, CST 2577), BRCA1 (1:200, Proteintech 22362-1-AP), PTEN (1:200, CST 9559), LKB1 (1:200, Santa Cruz sc-133742), Ki67 (1:1000, Abcam ab15580) and cleaved-Caspase 3 (1:300, CST 9661).^31, 37, 45, 47^ For each tumor sample, 3–5 random 40 × fields were scored. p-AKT, p-S6RP and p-4EBP1 protein levels were quantified using Image Pro Plus software.

### Genetically engineered mouse model and *in vivo* treatment studies

All animal procedures were conducted under the approval of the Animal Care and Use Committee at Dalian Medical University. At 8-week-old, female *Pten*^*loxp/loxp*^*/Lkb1*^*loxp/loxp*^ mice on FVB background were used for intrauterine injection with adenovirus expressing Cre recombinase to generate diseased mouse models with *Pten/Lkb1*-deficient endometrioid endometrial tumors as described previously.^37^ For drug treatment, Olaparib was dissolved in 10% hydroxypropyl-β-Cyclodextrin and dosed at 50 mg/kg per day (i.p.). BKM120 was dissolved in 0.5% methylcellulose and dosed at 30 mg/kg per day (p.o.).

### MRI analysis

MRI was used to assess treatment response *in vivo*. Mice were anesthetized with oxygen and 1% isoflurane gas and MRI was acquired on a MiniMR-RAT scanner (Niumag Electric Corporation, Shanghai, China). T2 weighted acquisitions were performed at day 0 (before treatment) and day 21 (at the end of treatment). Tumor volume calculations were performed for individual mice with Image J software (NIH) and presented in waterfall plots.

### Statistical analysis

Quantitative results were analyzed with Student’s *t* test. *P*-value <0.05 was considered as statistical significance.
