# Supplementary material for: PTEN deficiency sensitizes endometrioid endometrial cancer to compound PARP-PI3K inhibition but not PARP inhibition as monotherapy
Source: Oncogene. 2017 Sep 25;37(3):341–51. doi: 10.1038/onc.2017.326 (PMC5799770; doi:10.1038/onc.2017.326)
Supplement: Supplementary Figure [file onc2017326x1.pdf]

Supplementary Figure 1

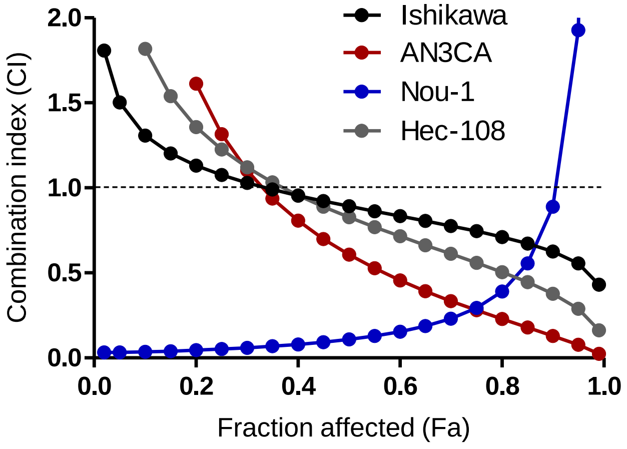

Supplementary Figure 1. The synergistic effect of PARP inhibitor Olaparib and PI3K inhibitor BKM120 in PTEN-deficient endometrioid endometrial cancer cells. Four PTEN-deficient endometrioid endometrial cancer cell lines were treated with Olaparib and BKM120 as single-agents or in combination for 72 hours and then subjected to CCK8 assay. Combination index (CI) values were determined using the established method of Chou and Talalay (CalcuSyn software).

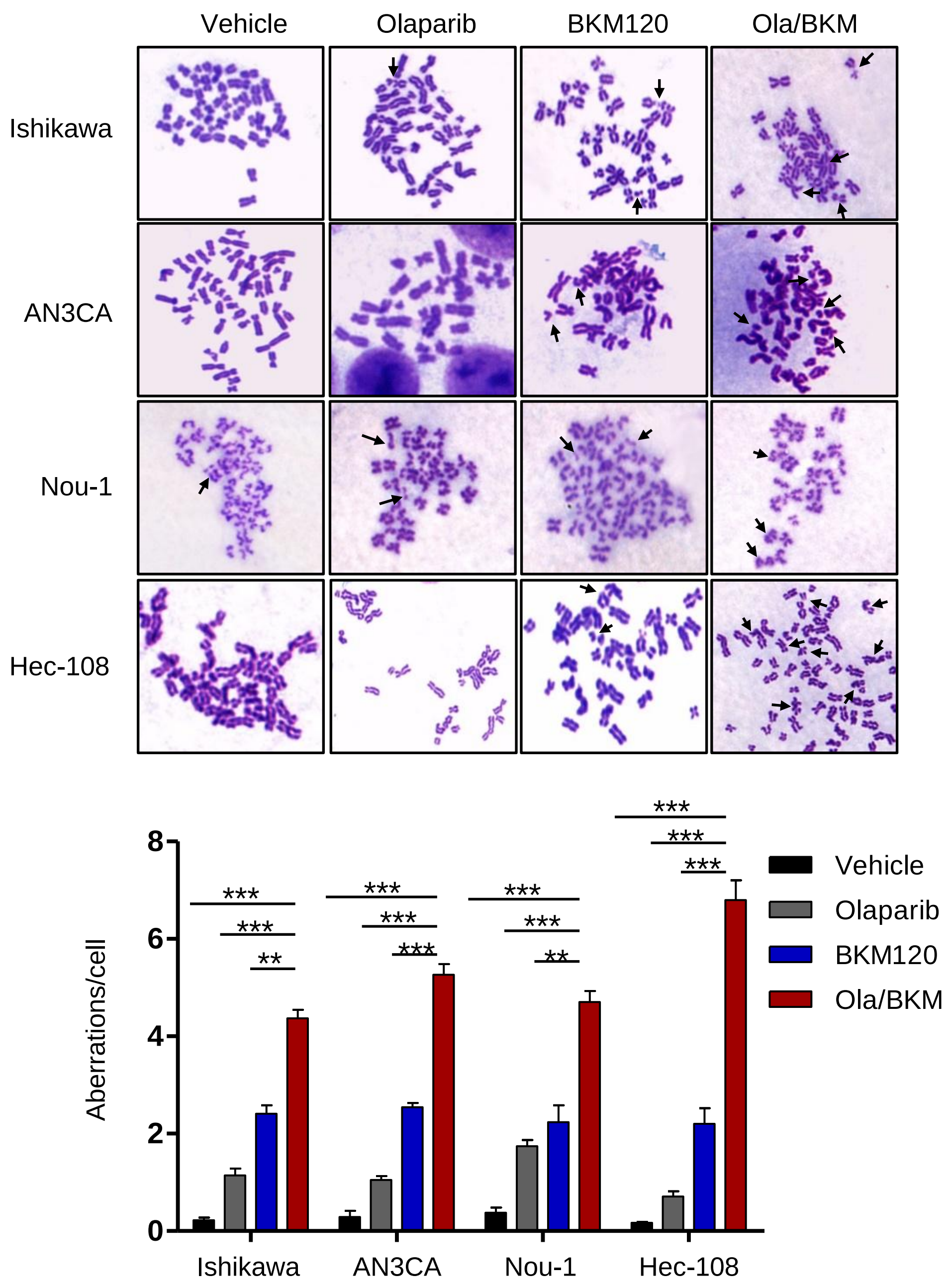

Supplementary Figure 2. Metaphase spread analysis of chromosome aberrations in four PTEN-deficient endometrioid endometrial cancer cells treated as indicated for 48 hours. Representative metaphase spreads are shown. Arrows indicate chromosomal aberrations. Mean ± S.D. for three independent experiments are shown. **P < 0.01; ***P < 0.001 (Student's t test).

Supplementary Figure 3

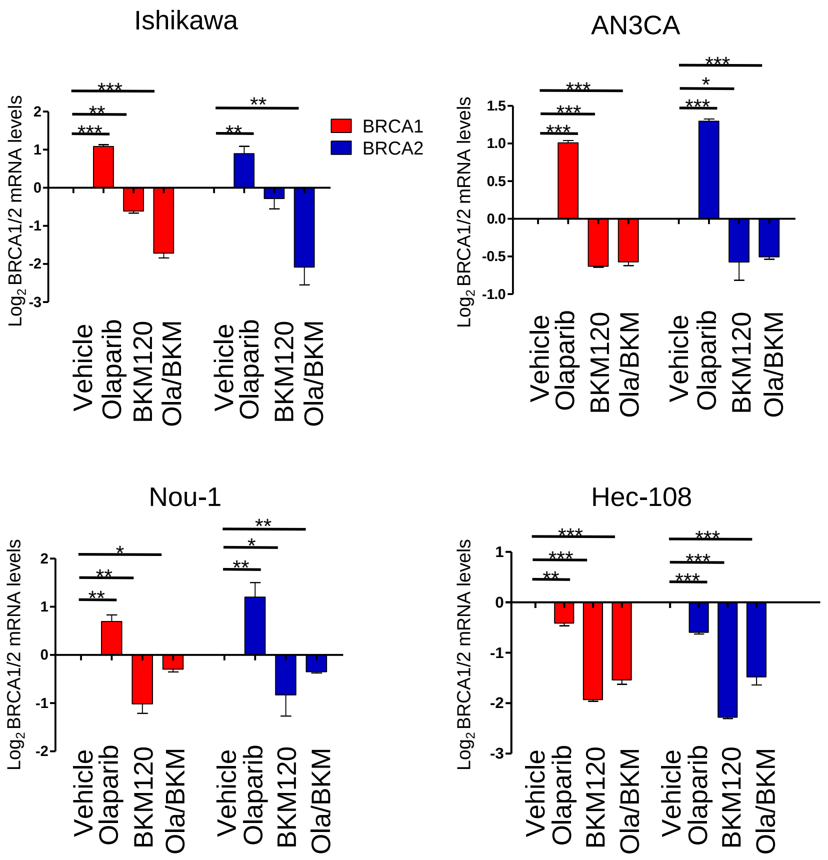

Supplementary Figure 3. Quantitative RT-PCR analysis of BRCA1 and BRCA2 expression in four PTEN-deficient endometrioid endometrial cancer cells treated as indicated for 24 hours. Gene expression was normalized to β-actin. Error bars represent mean ± S.D. These data are representative of three independent experiments. *P < 0.05; **P < 0.01; ***P < 0.001 (Student's t test).

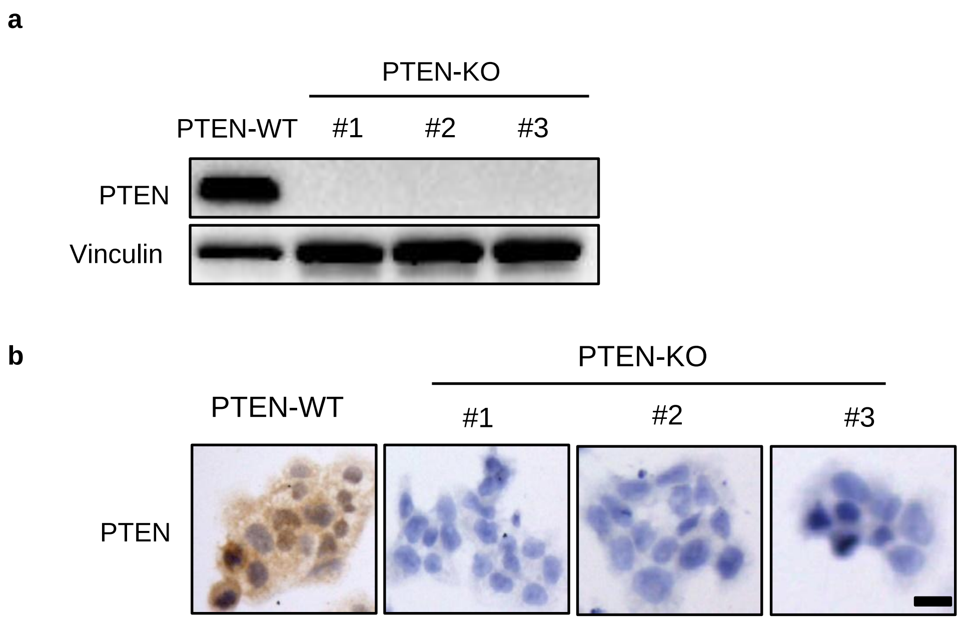

Supplementary Figure 4. PTEN expression analyses in Hec1-A endometrioid endometrial cancer cells. (**a**) Western blot analysis of PTEN in PTEN-proficient (PTEN-WT) and deficient (PTEN-KO) Hec-1A endometrioid endometrial cancer cells**.** Vinculin was used as a loading control. (**b**) Representative images of immunocytochemical staining analysis of PTEN in PTEN-proficient (PTEN-WT) and deficient (PTEN-KO) Hec-1A endometrioid endometrial cancer cells. Scale bar, 50 µm.

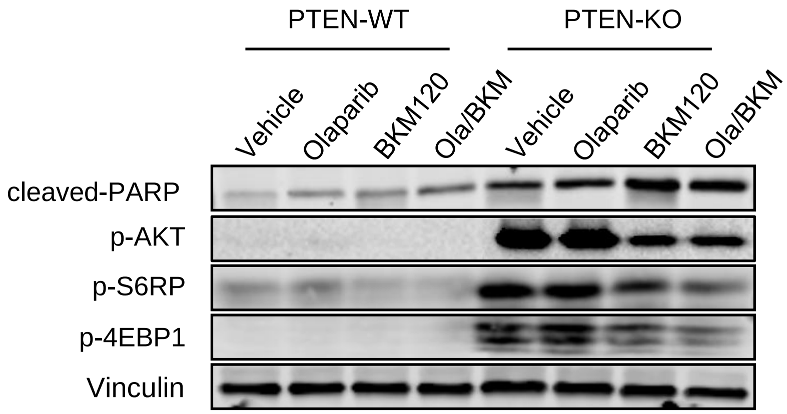

Supplementary Figure 5. Western blot analysis of proteins as indicated in PTEN-proficient (PTEN-WT) and deficient (PTEN-KO #3) Hec-1A endometrioid endometrial cancer cells treated with Olaparib and BKM120 as single-agents or in combination for 24 hours. Vinculin was used as a loading control.

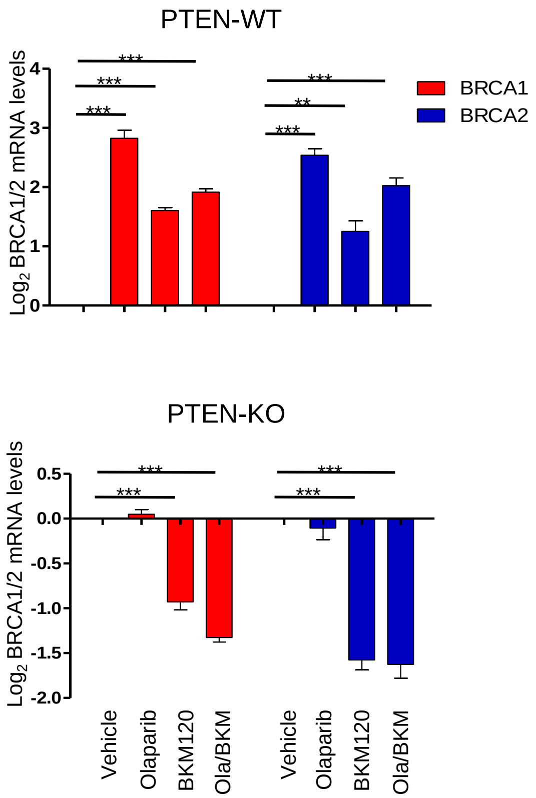

Supplementary Figure 6. Quantitative RT-PCR analysis of BRCA1 and BRCA2 mRNA expression in PTEN-proficient (PTEN-WT) and deficient (PTEN-KO #3) Hec-1A endometrioid endometrial cancer cells treated as indicated for 24 hours. Gene expression was normalized to β-actin. Error bars represent mean ± S.D. These data are representative of three independent experiments. $^{**}P < 0.01$; $^{***}P < 0.001$ (Student's *t* test).

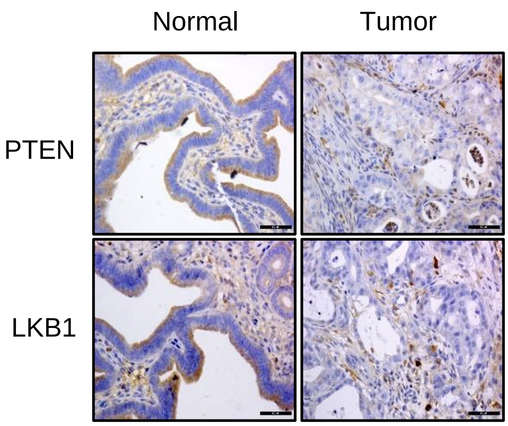

Supplementary Figure 7. Representative images of immunohistochemical staining of PTEN and LKB1 proteins in *Pten/Lkb1*-deficient endometrioid endometrial tumors (right panels). Scale bar, 50 μm. Left panels, normal uterus.

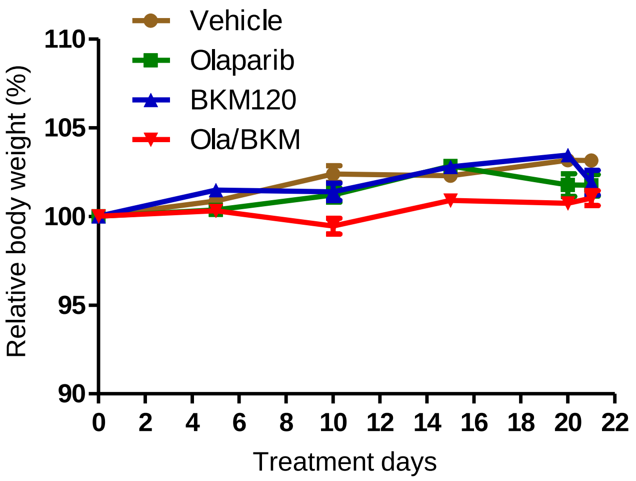

Supplementary Figure 8. Changes in body weight of Ade-Cre injected *Pten*$^{loxp/loxp}$/*Lkb1*$^{loxp/loxp}$ mice during 21 days treatments of Olaparib (50 mg/kg/day, intraperitoneal injection) and BKM120 (30 mg/kg/day, oral gavage) as single-agents or in combination. Error bars represent mean ± S.E.M. (n = 6-7 per treatment group).

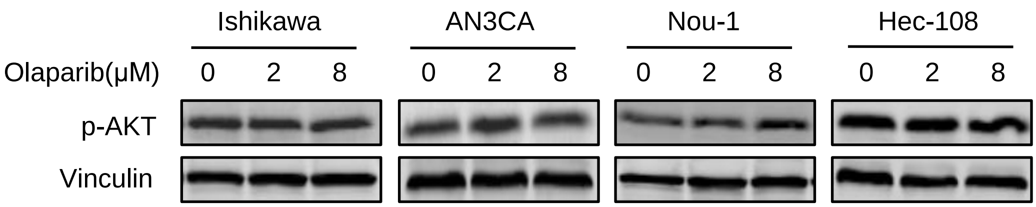

Supplementary Figure 9. Western blot analysis of p-AKT in four PTEN-deficient endometrioid endometrial cancer cell lines treated with Olaparib for 24 hours. Vinculin was used as a loading control.

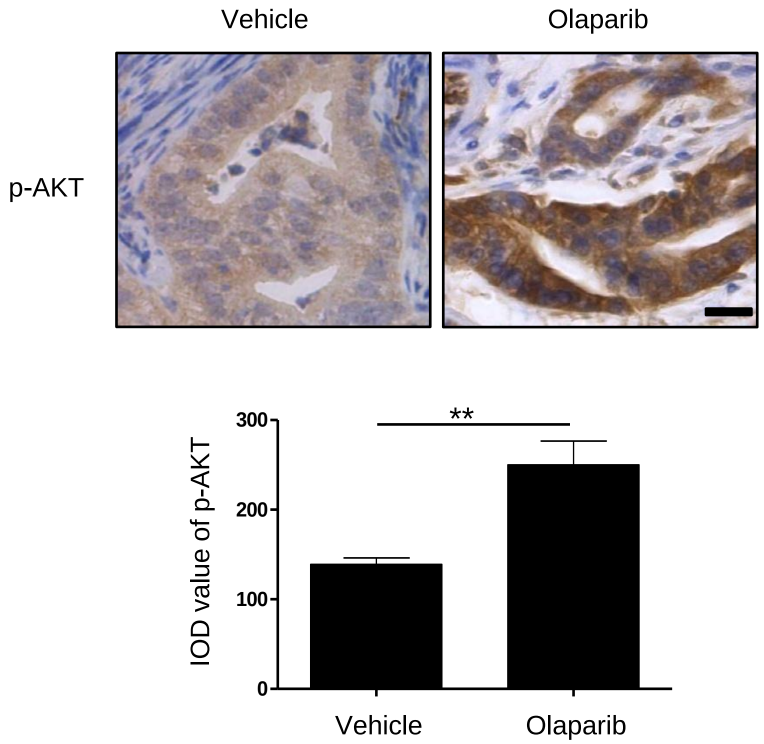

Supplementary Figure 10. Immunohistochemical staining of p-AKT in *Pten/Lkb1*-deficient endometrioid endometrial tumors. Representative images of immunohistochemical staining from Ade-Cre injected *Pten*$^{loxp/loxp}$/*Lkb1*$^{loxp/loxp}$ mice treated with Olaparib (50 mg/kg/day, intraperitoneal injection) for 10 days (right panel). Scale bar, 25μm. Tumor treated with vehicle was used as a control (left panel). Quantification of IOD value is shown (bottom panel). Error bars represent mean ± S.E.M. (n = 6 per group). **P < 0.01 (Student's *t* test).
